# Functions of nitroreductases in mycobacterial physiology and drug susceptibility

**DOI:** 10.1128/jb.00326-24

**Published:** 2025-01-08

**Authors:** Ifeanyichukwu E. Eke, Robert B. Abramovitch

**Affiliations:** 1 Department of Microbiology, Genetics & Immunology, Michigan State University 3078 , East Lansing, Michigan, USA; University of Notre Dame, Notre Dame, Indiana, USA

**Keywords:** *Mycobacterium*, nitroreductase, antimicrobials

## Abstract

Tuberculosis is a respiratory infection that is caused by members of the *Mycobacterium tuberculosis* complex, with *M. tuberculosis* (Mtb) being the predominant cause of the disease in humans. The approval of pretomanid and delamanid, two nitroimidazole-based compounds, for the treatment of tuberculosis encourages the development of more nitro-containing drugs that target Mtb. Similar to the nitroimidazoles, many antimycobacterial nitro-containing scaffolds are prodrugs that require reductive activation into metabolites that inhibit the growth of the pathogen. This reductive activation is mediated by mycobacterial nitroreductases, leading to the hypothesis that these nitroreductases contribute to the specificity of the nitro prodrugs for mycobacteria. In addition to their prodrug-activating activities, these nitroreductases have different native activities that support the growth of the bacteria. This review summarizes the activities of different mycobacterial nitroreductases with respect to their activation of different nitro prodrugs and highlights their physiological functions in the bacteria.

## INTRODUCTION

Tuberculosis (TB) is a respiratory infection that is caused by a phylogenetically related group of species known as the *Mycobacterium tuberculosis* complex (MTBC). Members of this complex include *Mycobacterium tuberculosis* (Mtb), *Mycobacterium africanum*, *Mycobacterium bovis*, *Mycobacterium orygis*, and *Mycobacterium canettii* among others, with Mtb being the predominant cause of TB in humans. With the introduction of antimycobacterial drugs such as streptomycin, isoniazid, rifampicin, pyrazinamide, and ethambutol, the 20th century gave birth to modern TB chemotherapy. While the latter four drugs remain the standards for TB treatment, their effectiveness is hampered by the evolution and spread of drug-resistant strains. Additionally, TB treatment with these drugs is limited by the long courses of treatment needed to effectively sterilize the body of the pathogen (1–12). These challenges require the discovery and development of new drugs to treat TB.

Nitric oxide is part of the body’s innate immune system against mycobacterial infections (1, 13, 14). Not surprisingly, nitro-containing compounds have emerged as important additions to the TB drug repository (1, 3, 8, 15). Many nitro-containing compounds are prodrugs, requiring the reductive activation of their pharmacophoric nitro groups in order to exert their antimycobacterial activities (6, 16). The reduction of the nitro prodrugs is usually mediated by cofactor-dependent mycobacterial nitroreductases, making these compounds specific for mycobacteria.

**Fig 1:**
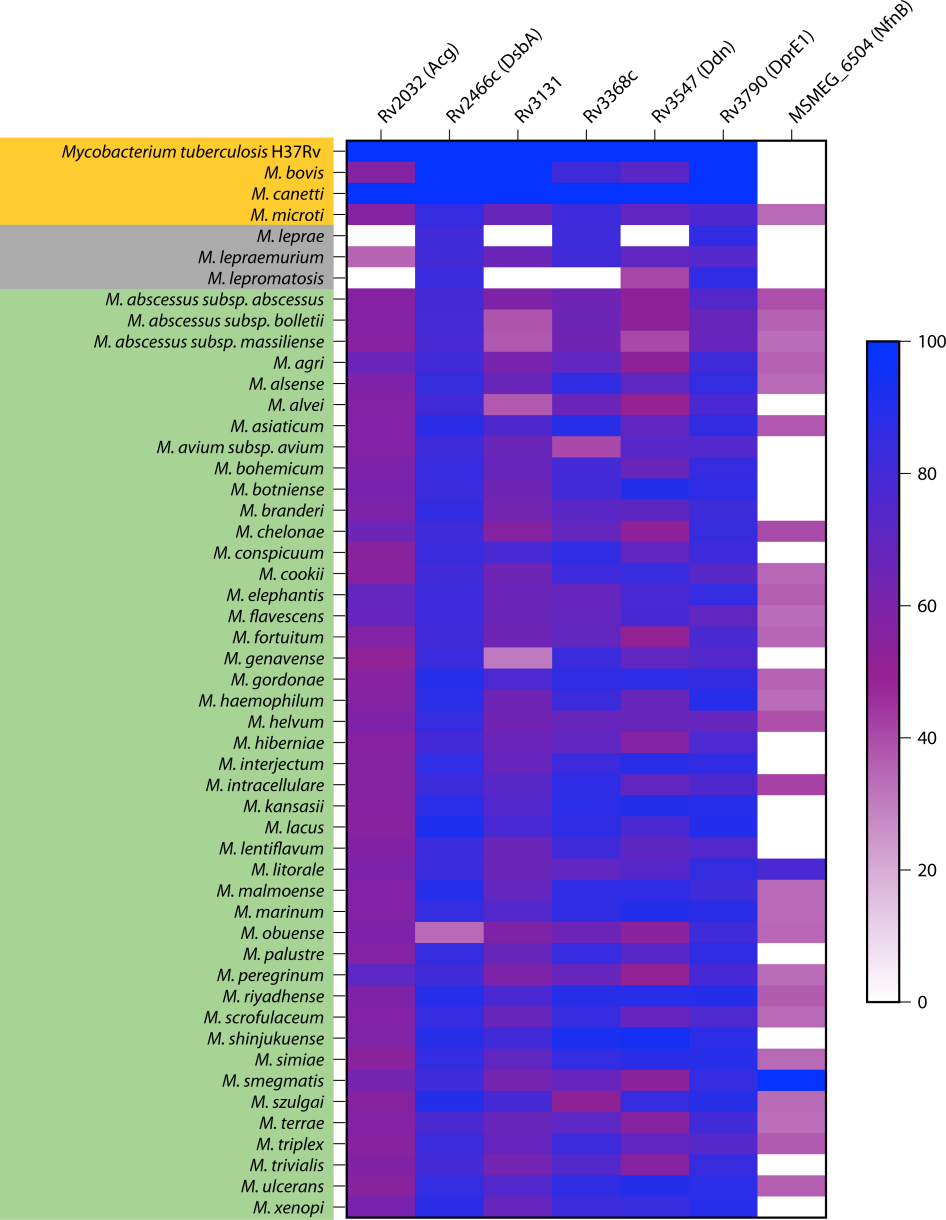
The conservation of mycobacterial nitroreductases across different species. The color gradient is the percentage homology of the amino acid sequences of the enzymes with respect to the *M. tuberculosis* H37Rv homolog (for Rv2032, Rv2466c, Rv3131, Rv3368c, Rv3547, and Rv3790) or *M. smegmatis* MC^2^-155 homolog (for MSMEG_6504). Homologs were determined using a threshold e-value of 1 × 10^−14^. The color shade for each species represents the type of infection caused, yellow/tuberculosis, gray/leprosy, and green/nontuberculous mycobacterial infection.

One of these mycobacterial nitroreductases is the deazaflavin-dependent nitroreductase (Ddn). Much is known about Ddn because of its role as the primary nitroreductase involved in the activation of pretomanid and delamanid, two nitro-containing drugs that have been approved for TB treatment (1, 3, 6–9, 17). There are also other mycobacterial nitroreductases such as NfnB, Acg, DsbA, DprE1, Rv3368c, and Rv3131 (Fig. 1), and they activate different nitro-containing scaffolds (Fig. S1). In this review, our emphasis is placed on discussing the mechanistic basis for the prodrug-activating activities of the nitroreductases. Where it is known, the native activity of the nitroreductases is also discussed, and gaps in our understanding of the systems are highlighted.

## DEAZAFLAVIN-DEPENDENT NITROREDUCTASE (Ddn/Rv3547)

### Native function of Ddn

**Fig 2:**
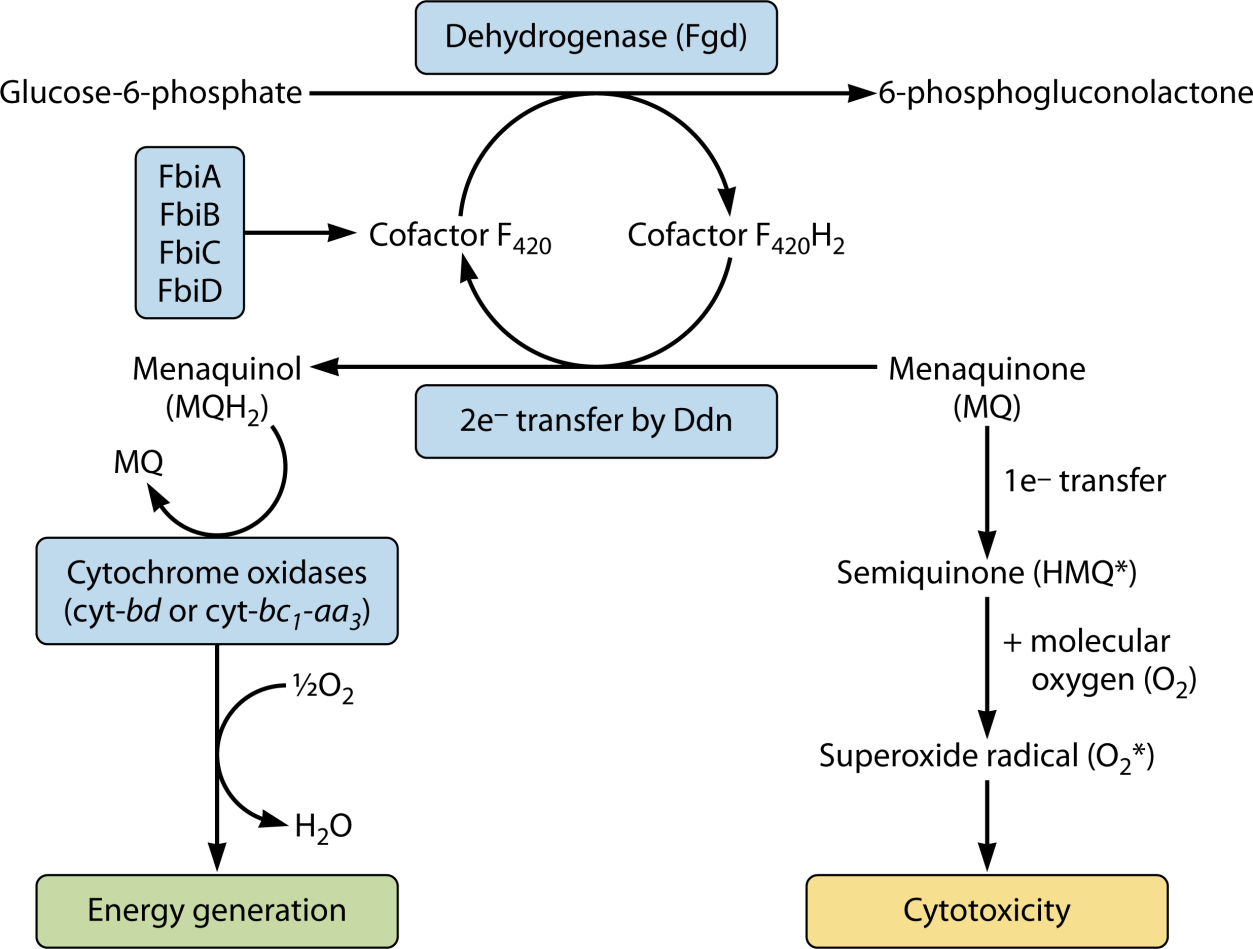
Schematic on the native activity of Ddn. First, FbiA, FbiB, FbiC, and FbiD work together in the biosynthesis of cofactor F_420_. This oxidized cofactor can be converted into the reduced form through the activity of Fgd in the pentose phosphate pathway. The reduced cofactor can be used by Ddn to reduce menaquinone into the menaquinol form in a two-electron transfer. Menaquinol can subsequently transfer electrons to the terminal cytochrome *bd* or *bc*_*1*_*-aa*_*3*_ oxidases, leading to the reduction of oxygen and energy production. Alternatively, in the absence of the menaquinone-reductase activity of Ddn, menaquinone is reduced in a one-electron transfer to unstable semiquinone that can react with molecular oxygen to form superoxide radicals, leading to the death of the cells.

Ddn (Rv3547) and its homologs (Rv1558, Rv1261c, and Rv3178) are classified as F_420_H_2_-dependent quinone reductases because of their dependence on cofactor F_420_ to reduce different quinone-based substrates such as menaquinone, menadione, and plumbagin (18). However, it is the reduction of menaquinone that is physiologically most relevant (17, 18), and accordingly, Ddn is annotated as an F_420_H_2_-dependent menaquinone reductase (17). In the mycobacterial electron transport system, menaquinone serves as an essential intermediate that shuttles electrons from the membrane-bound NADH dehydrogenases and succinate dehydrogenases to the cytochrome complexes (cyt-*bc_1_-aa_3_* or cyt*-bd*). The shuttled electrons can then be used to reduce oxygen or other terminal electron acceptors, producing a proton motive force that is used to power ATP generation (19). The menaquinone reductase activity of Ddn has been associated with energy generation (17), where Ddn is proposed to use F_420_H_2_ as a respiratory electron source to reduce menaquinone into menaquinol (Fig. 2). The reduced menaquinone donates its electrons to cytochrome complexes (cyt-*bc_1_-aa_3_* or cyt*-bd*), with the subsequent activity of the cytochromes leading to oxygen reduction and ATP production. This model was built from data generated from purified membrane fractions (17) and needs to be verified using orthogonal approaches in intact cells. Nevertheless, F_420_H_2_ may be considered as part of the mycobacterial pool of respiratory electron donors. This might also provide metabolic flexibility in hypoxic and stressful conditions, where F_420_H_2_, through menaquinol, can contribute electrons that are used by the cytochrome complexes in the respiratory reduction of oxygen (19–21). Therefore, the menaquinone-reductase activity of Ddn coupled with the activity of cytochrome complexes may help in maintaining the proton motive force in non-replicating persistent Mtb, a hypothesis that still needs to be tested.

In addition to respiration, the quinone reductase activity of Ddn has been linked to resistance against oxidative stress in mycobacteria (17, 18, 22, 23). Guerra-Lopez et al. reported that *M. smegmatis* mutants deficient in cofactor F_420_ biosynthesis are more sensitive to quinone-based oxidative stress agents (22), a finding that was also replicated in F_420_-deficient Mtb strains (18). Hasan et al. observed a reduction in the intracellular levels of glucose-6-phosphate in mycobacterial cells that were challenged with quinone-based oxidative stress agents, and the disruption of the F_420_-dependent glucose-6-phosphate dehydrogenase (*fgd*) made the cells more sensitive to these agents (23). This finding supports the hypothesis that glucose-6-phosphate and F_420_H_2_ function as electron storage molecules that maintain the redox balance of the cell during oxidative stress. However, when oxidative stress agents were introduced into a cell lysate containing F_420_H_2_, there was no observable reduction of these agents. Considering this, it was speculated that the protective effects conferred by the reducing power of F_420_H_2_ might be occurring through an enzyme intermediate that was not in sufficient amount in the cell lysate. Indeed, this was later found to be true when Gurumurthy et al. showed that Ddn uses F_420_H_2_ to reduce different quinone substrates (18). Interestingly, reducing cofactors such as NADH or NADPH are not used by the enzyme, nor does it depend on metal ions for its activity (2, 18). Gurumurthy et al. proposed a Ddn-dependent oxidative stress resistance where Ddn, in a two-electron transfer, uses F_420_H_2_ to reduce quinones such as menaquinone into the quinol form (18) (Fig. 2). This two-electron transfer by Ddn competes with the toxic one-electron reduction of quinones that normally leads to the generation of the unstable semiquinone molecule. While quinols can easily be detoxified, the semiquinones reacts with molecular oxygen to form superoxide radicals that kill the cells. Therefore, in mediating the conversion of menaquinone into quinols instead of semiquinones, the menaquinone-reductase activity of Ddn is protecting against oxidative stress. Despite the plausibility of this model, it needs to be further tested since quinols can also be oxidized to cytotoxic semiquinones (18).

Lastly, the F_420_H_2_-dependent quinone reductase activity of Ddn and its homologs can be considered as an important recycling system to regenerate the oxidized cofactor that is used by Fgd in the pentose phosphate pathway (PPP) to oxidize glucose-6-phosphate into phosphogluconate. Of note, the oxidized cofactor can also be used by a structural homolog of Fgd, Rv0132c, initially annotated as Fgd2 (24, 25). Rv0132c is not a glucose-6-phosphate dehydrogenase since it cannot oxidize glucose-6-phosphate (24, 25). However, it is involved in the biosynthesis of mycolic acids where it uses cofactor F_420_ in the oxidation of hydroxy-mycolic acid into keto-mycolic acid (24). Therefore, Rv0132c has been re-annotated as F_420_-dependent hydroxy mycolic acid dehydrogenase (FHMAD) (24, 26). Both Fgd and FHMAD utilize cofactor F_420_ to catalyze different reactions, producing F_420_H_2_; however, Fgd is the primary source of F_420_H_2_ since many F_420_H_2_-dependent reactions cannot occur when Fgd is genetically ablated (26). A possible explanation for this is that the Fgd-linked PPP is a biochemical pathway that is required throughout the lifecycle of the bacteria, serving as a source of ribose sugars and reducing equivalents such as F_420_H_2_. This contrasts with the FHMAD-linked generation of F_420_H_2_ that occurs only during mycolic acid synthesis in preparation for cellular division. In any case, the F_420_H_2_ produced by either enzyme needs to be recycled into an oxidized form that can be reused. This is where Ddn comes into play to regenerate the oxidized cofactor. In addition to Ddn, it is important to note that Rv2951c, a phthiodiolone ketoreductase, can also recycle F_420_H_2_ into an oxidized form through its participation in the biosynthesis of phthiocerol dimycocerosates (26). The same is applicable to Rv2074, an F_420_H_2_-dependent biliverdin reductase, that uses F_420_H_2_ to reduce heme-derived biliverdin into bilirubin (27). Therefore, Ddn, Rv2951c, and Rv2074 are some of the few reductases that recycle F_420_ into an oxidized form. It can be argued that without the reductase activity of these enzymes, the cell would always turn to the metabolically burdensome *de novo* biosynthesis of cofactor F_420_ to satisfy its need for the oxidized form that is needed by Fgd and FHMAD. Taken together, Ddn is proposed to be part of the energy-generating machinery and oxidative stress defense system of mycobacteria.

## PRODRUG-ACTIVATING ACTIVITY OF Ddn

**Fig 3:**
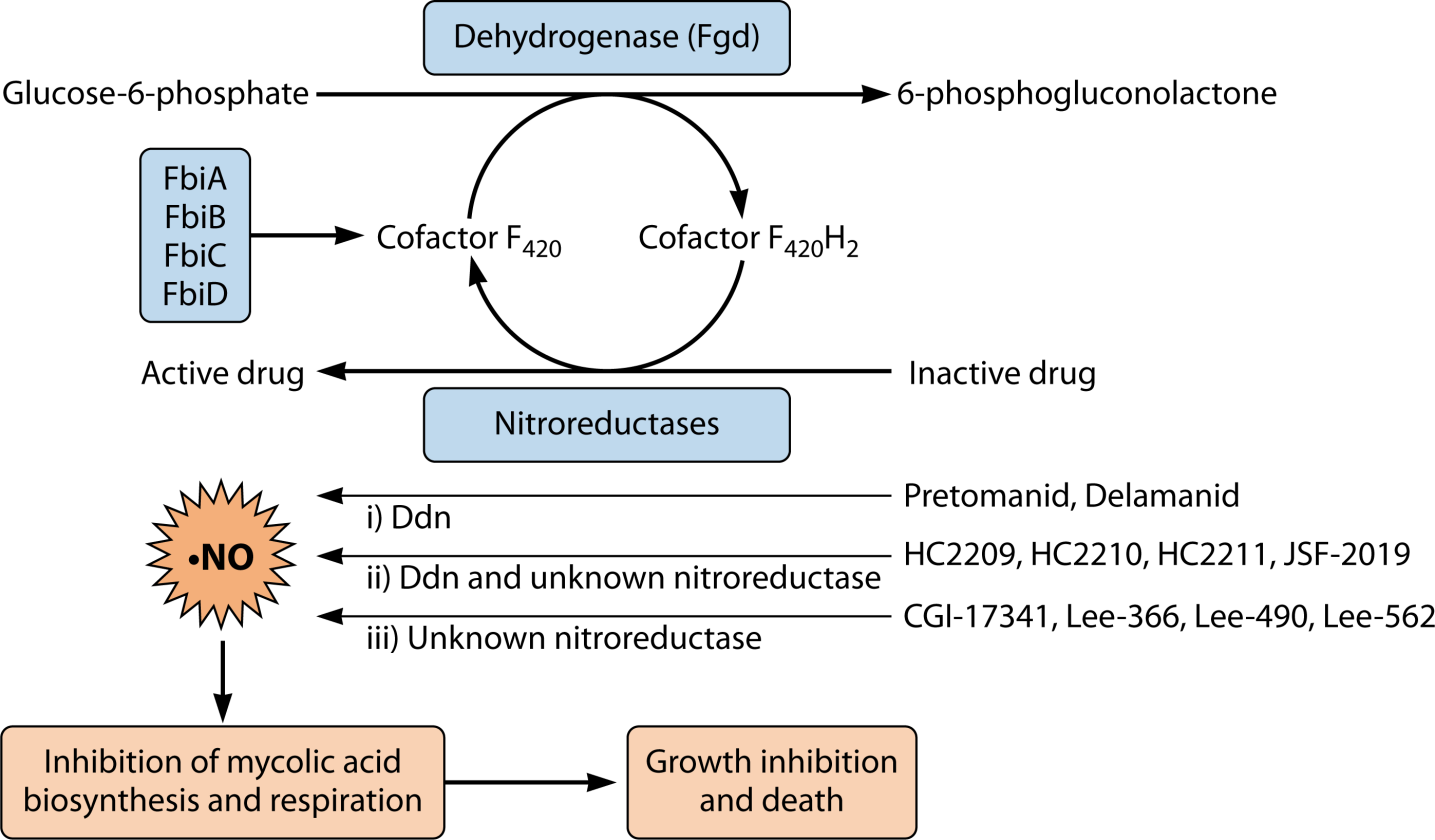
Schematic on the activation of different F_420_-dependent compounds. The reduced cofactor F_420_, that is produced by Fgd, is used by nitroreductases to reductively activate different nitro-containing compounds. Ddn is the exclusive nitroreductase for pretomanid and delamanid. HC2209, HC2210, HC2211, and JSF-2019 depend on Ddn and another F_420_-dependent nitroreductase that is unknown. CGI-17341, Lee-366, Lee-490, and Lee-562 need the reduced cofactor F_420_ for their activation, although the utilizing nitroreductase is yet to be discovered. Upon reductive activation, nitric oxide species are proposed to be produced, and these inhibit the biosynthesis of mycolic acid and poison the electron transport chain, leading to growth inhibition and death.

Ddn is commonly known for its role as the nitroreductase required for the reductive bioactivation of the nitroimidazole-based TB drugs, pretomanid and delamanid (1, 3, 6–8, 16, 17). However, other categories of cofactor F_420_-dependent nitro compounds have been reported (Fig. 3). For example, there are those that require Ddn and possibly another F_420_-dependent nitroreductase for their activity. These include the nitrofuranyl piperazines (HC2209, HC2210, and HC2211) (15) and the nitrofuranyl triazines (9). Then, there are those that depend on cofactor F_420_ but not Ddn for their activation. For such compounds, the activating nitroreductase is yet to be identified, but it is predicted that the enzyme must depend on cofactor F_420_ for its activity. A good example is CGI-17341, the parent analog for pretomanid and delamanid, that retains its activity against *ddn* mutants (6). Intriguingly, while CGI-17341 is able to inhibit the growth of *ddn* mutants (2, 6), *in vitro* biochemical characterization shows that Ddn can reduce the compound albeit at a lower rate compared to pretomanid (2). This suggests that redundant Ddn homologs might be playing a role in its activation inside the cell. Another example is the nitrofuranylamides that retain their antimycobacterial activity in the absence of Ddn, but lose it in the absence of cofactor F_420_ or *fgd* (16).

Ddn can reductively activate a variety of nitro prodrugs, and a detailed biochemical and structural basis for this reductive activation is best described for pretomanid. The crystal structure of Ddn was solved by Cellitti et al. (1), but the generation of a cocrystal structure of Ddn and pretomanid has yet to be reported (1, 17). Considering this limitation, different molecular docking tools have been used to model the interactions of pretomanid with the protein (1, 2, 28). The Ddn structure has a split barrel-like topology and a positively charged groove that interacts with the complementary negatively charged oligoglutamyl tail of cofactor F_420_ (1). These interactions occur through a network of salt bridges and hydrogen bonding. In addition to the oligoglutamyl tail, different components of the cofactor such as the phosphate group, the ribityl moiety, and the deazaflavin ring interact with the enzyme to stabilize the binding of the cofactor. On the side of the enzyme, residues such as R54, K55, T56, R60, N62, P63, Y65, A76, S78, K79, M87, W88, N91, and Y133 among others participate in these interactions. Additionally, water molecules can mediate some interactions between the enzyme and the cofactor. Cellitti et al. proposed that a combination of all these interactions leads to the orientation of the *Re* face of cofactor F_420_ toward pretomanid (1). Predictably, resistance to pretomanid and other Ddn-dependent compounds has been linked to mutations in these residues (1, 6, 15, 17, 28). The *Re* orientation of F_420_ for pretomanid activation in Ddn contrasts with Fgd and many other F_420_-dependent enzymes that catalyzes reactions at the *Si* face of the cofactor (1). The *Re* and *Si* nomenclature of a molecule describes the three-dimensional positioning of groups around a prochiral center, with the clockwise and anti-clockwise orientations of the groups representing the *Re* and *Si* faces, respectively. These three-dimensional arrangements in space influence many biological reactions due to the stereospecificity of many enzymes.

In the molecular docking of pretomanid to Ddn, the drug is first placed in the protein such that its nitroimidazole group is close to the carbon-5 of the deazaflavin ring of cofactor F_420_ (1). Upon docking to Ddn, the nitroimidazole group of pretomanid is positioned near the *Re* face of cofactor F_420_ and the hydrophobic tail of the drug is oriented toward the N-terminus of the protein (1, 2). Additionally, the nitro group of pretomanid interacts with S78, Y130, and Y136 through hydrogen bonding (1, 17). While it is tempting to suggest that Ddn directly participates in the transfer of the hydride electrons from the cofactor to the drug, the absence of classic catalytic residues at the active site of Ddn argues against this possibility (1). In this case, Ddn functions primarily by precisely positioning the nitroimidazole head group of pretomanid near the *Re* face of F_420_ for an efficient hydride transfer from F_420_ and possibly aiding in the stabilization of the transition state of the drug-F_420_ complex (1). Subsequently, the electron-deficient imidazole group of pretomanid is subject to a hydride attack from the deazaflavin ring of cofactor F_420_ (2, 7). This attack occurs at the carbon-3 position of the imidazole ring, leading to the reduction of the ring and a concomitant formation of three intermediates of the drug. One of these intermediates further decomposes to release a mycobactericidal burst of nitric oxide (2, 7, 29).

## CONSERVATION OF Ddn

Ddn is highly conserved across many mycobacterial species but is lacking in *M. leprae* (Fig. 1). Mutants deficient in F_420_ biosynthesis and *ddn* mutants do not show any growth defect under standard laboratory growth conditions (18, 28, 30), suggesting it is not essential for growth. There are varying numbers of Ddn homologs in different mycobacterial species (1, 6, 17, 18, 28), with three in Mtb and as many as 11 in *M. abscessus*. Remarkably, none of the three homologs in Mtb has been shown to be involved in the activation of any prodrug nor can they serve as a replacement for Ddn in the activation of pretomanid and delamanid. Nonetheless, these homologs have quinone reductase activity like Ddn and are part of the defense system against oxidative stress (18).

Some mycobacterial species that have a Ddn ortholog are unable to activate pretomanid and delamanid, possibly due to differences in the key residues that interact with the drug or cofactor or, alternatively, differences in metabolism of the agents, or permeability or efflux of the compounds across the mycomembrane (1, 17, 31, 32). For example, the Ddn ortholog of Mtb and *M. marinum* shares a high similarity of essential residues necessary for prodrug activation (Fig. S2), making both species susceptible to pretomanid and delamanid treatment (17, 33, 34). *M. kansasii* and *M. xenopi* are also susceptible to pretomanid (33, 34). However, *M. smegmatis*, *M. ulcerans*, *M. avium, M. intracellulare*, *M. gordonae*, *M. chelonae*, *M. fortuitum*, *M. scrofulaceum*, *M. gilvum*, and *M. abscessus* are resistant to pretomanid (6, 17, 33–35). In our recently published work (15), we found that both pretomanid and the nitrofuranyl piperazines (HC2209, HC2210, and HC2211) are active against Mtb, but only the latter three retain their activity against *M. abscessus*. Pretomanid depends exclusively on Ddn for activation, while the nitrofurans depend on Ddn and possibly another F_420_-dependent nitroreductase for their activation. It remains to be determined if these differences in specificity are driven by interactions of the compounds with Ddn or other indirect effects such as permeability or efflux across the mycomembrane.

## COFACTOR F_420_ AND ITS BIOSYNTHESIS

Cofactor F_420_ is so named because of the characteristic peak absorbance of its oxidized state at 420 nm (8, 30, 36). It is a deazaflavin-based molecule that is conjugated to an oligoglutamyl tail of varying lengths. Structurally, cofactor F_420_ is similar to riboflavin cofactors such as flavin adenine dinucleotide (FAD) and flavin mononucleotide (FMN), but biochemically, it functions more like the nicotinamides such as NAD and NADP (29, 30, 36, 37). It is an obligatory redox carrier of two electrons and participates in hydride transfer. It has a low standard redox potential range of −340 mV to −360 mV, compared to −205 mV to −220 mV for the flavins and −320 mV for nicotinamides (24, 30, 36). The lower redox potential of cofactor F_420_ translates to a stronger reducing power and enables it to reduce a variety of substrates (1, 2, 18, 30). This property has been proposed to allow the cofactor to serve as an electron carrier in low-oxygen or highly anaerobic environments (6, 24, 30). This biochemical property was initially used to explain the presumed taxonomical restriction of the cofactor to few groups such as archaea and actinobacteria where they participate in metabolically challenging transformations such as methanogenesis and sulfate reduction (30). However, evidence has recently arisen on the possible widespread distribution of the cofactor in non-actinobacterial phyla, although the physiological role of the molecule in these bacteria is still unclear (36).

The biosynthesis of cofactor F_420_ is a multi-enzymatic process that involves precursor molecules such as phosphoenolpyruvate, GTP, deazaflavin (F_0_), lactate, tyrosine, and glutamate among others (8, 28, 30, 37). A detailed review of the biosynthesis of cofactor F_420_ was provided by Greening et al. (30). First, F_0_, a biosynthetic intermediate of cofactor F_420_, is produced through the condensation activity of F_0_ synthase using precursors such as tyrosine and the pyrimidine, ribityldiaminouracil. In Mtb, this synthase is a single protein (FbiC), but in archaea, it is composed of two proteins (CofG and CofH). Next, enzymes such as CofA, CofB, and CofC (FbiD) work together to produce a lactate-derived intermediate that condenses with F_0_ to form F_420_-0. Recently, Bashiri et al. proposed a revision to this condensation reaction in prokaryotes where a phosphoenolpyruvate intermediate instead of a lactate-derived intermediate is involved (37). Either way, the condensation reaction is catalyzed by FbiA or CofD, and the final product, F_420_-0, is highly similar to cofactor F_420_ except that it lacks an oligoglutamyl tail. Lastly, cofactor F_420_ is generated through the sequential addition of glutamate residues to F_420_-0. This reaction occurs in a GTP-dependent manner and is catalyzed by an F_420_:γ-L-glutamyl ligase, CofE or FbiB. It should be noted that the genes involved in F_420_ biosynthesis seem to be functionally non-redundant in Mtb since the disruption of any of these genes leads to a halt in the biosynthesis of the cofactor and a concomitant resistance to different F_420_-dependent drugs (8, 28).

## OTHER MYCOBACTERIAL NITROREDUCTASES

### NfnB (MSMEG_6505)

NfnB is an FMN-dependent nitroreductase that uses NADPH or NADH in a double-displacement reaction to reduce nitro-containing substrates into different derivatives (38, 39). NfnB first reduces its prosthetic FMN group using NADPH or NADH as the electron source. Subsequently, it uses the reduced FMN to reduce the nitro groups of different nitro-aromatics, generating amino or hydroxylamino derivatives (38). As shown in Fig. 1, NfnB is lacking in many mycobacterial species including the MTBC. However, it is present in some fast-growing species, such as *M. smegmatis*, and much of what is currently known for mycobacterial NfnB came from the *M. smegmatis* homolog, *MSMEG_6505*.

The expression of MSMEG_6505 is controlled by the neighboring transcriptional repressor, MSMEG_6503, that binds to conserved operator sites in MSMEG_6505 and represses the expression of the enzyme (39). Genetic ablation of MSMEG_6503 leads to the overexpression of MSMEG_6505 (39–41). Many of the studies identifying MSMEG_6505 as a nitroreductase for different prodrugs resulted from forward genetic selections where the expression of MSMEG_6503 is disrupted (15, 39–41). None of these selections reported spontaneous mutations in MSMEG_6505, bringing up the question of why the regulator is easily disrupted in different genetic selection studies while the nitroreductase stays intact.

The native physiological activity of MSMEG_6505 remains enigmatic; hence, the protein has primarily been studied in the context of the modification of exogenous nitro-containing substrates. Unlike most mycobacterial nitroreductases that only have a prodrug-activating activity, MSMEG_6505 can activate or inactivate a nitro prodrug, and this seems to be dependent on the type of scaffold that is possessed by the nitro substrate (15, 39–41). For instance, benzothiazinones, nitroimidazoles, and dinitrobenzamides are modified by MSMEG_6505 into inactive amino or hydroxylamine derivatives (39, 40, 42, 43). We have also found that the dinitrobenzamides (HC2217 and HC2238) lose their activity against *MSMEG_6503* mutants, presumably due to the increased expression of MSMEG_6505 (15). In contrast, the nitazoxanides are reductively activated by the enzyme into toxic hydroxylamine intermediates that kill the bacteria (41). Additionally, overexpression of the enzyme in *M. smegmatis* increases the susceptibility of the bacteria to thienopyrimidines (44).

### DprE1 (Rv3790)

**Fig 4:**
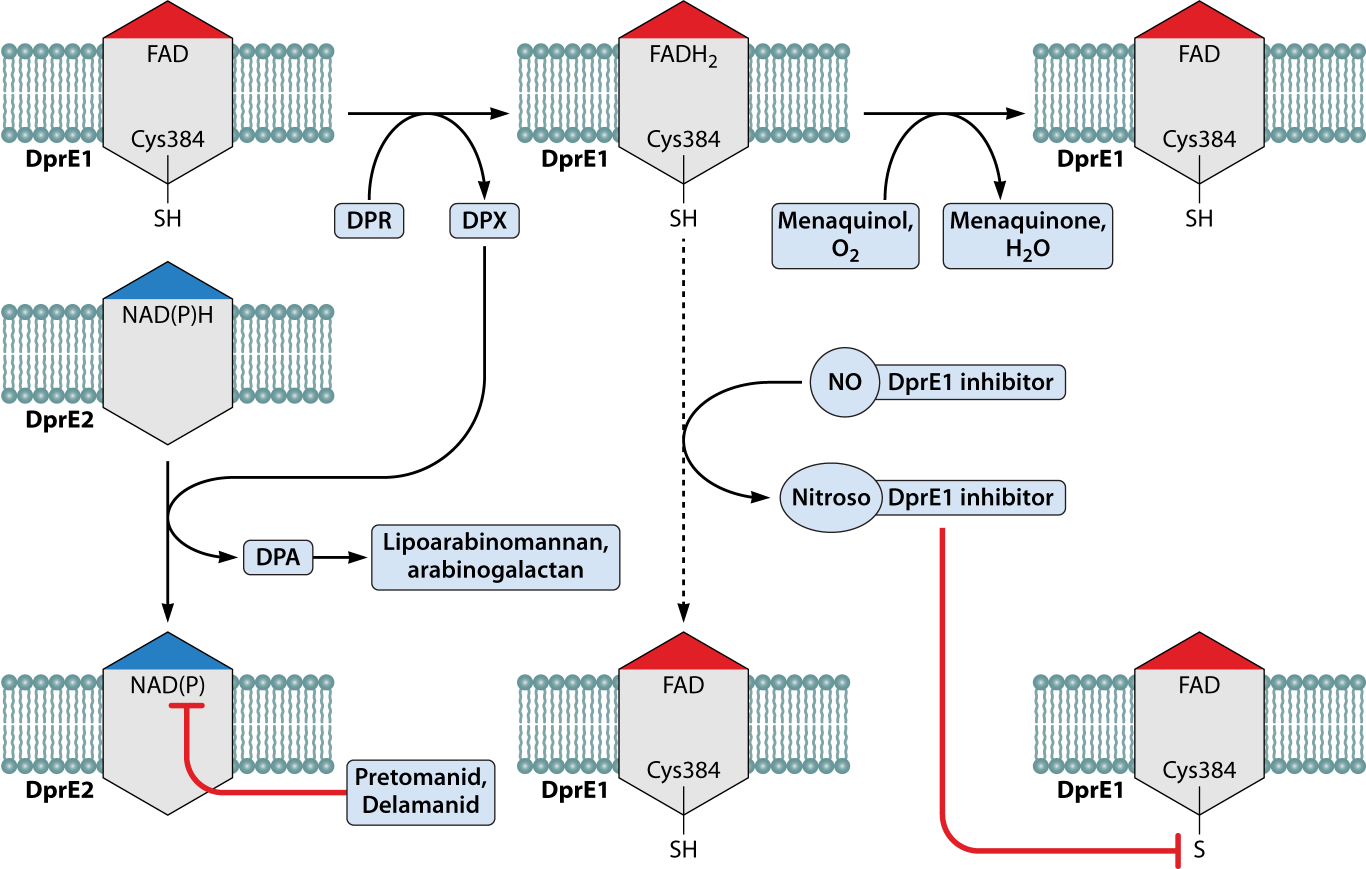
Activity of DprE1 mechanism-based inhibitors and DprE2 inhibitors. DPR (decaprenylphosphoryl-β-D-ribose) is converted to DPX (decaprenylphosphoryl-D-2-keto-ribose) through the catalytic activity of DprE1. This reaction produces the reduced form of the bound coenzyme, FADH_2_, and the oxidized coenzyme is regenerated through the oxidizing activities of menaquinol or molecular oxygen. However, nitro-containing DprE1 inhibitors can regenerate the bound oxidized coenzyme through a DprE1-mediated activity, forming a nitroso intermediate of the inhibitors. Subsequently, the nitroso intermediates form a covalent bond with the thiol group of Cys384 at the active site of DprE1. This covalent modification inhibits the activity of DprE1. The DPX, that is produced by DprE1, is converted to DPA through the activity of DrpE2. DPA is the sole source of arabinosyl groups that are used in the biosynthesis of the lipoarabinomannan and arabinogalactan components of the mycobacterial cell envelope. Pretomanid and delamanid inhibit the activity of DprE2 by forming an adduct with the protein.

DprE1, decaprenylphosphoryl-β-D-ribose oxidase/Rv3790, is a highly conserved protein required for the biosynthesis of components of the mycobacterial cell envelope (Fig. 1 and 4). DprE1 forms a heteromeric membrane-bound epimerase complex with DprE2 (decaprenylphosphoryl-D-2-keto-ribose reductase/Rv3791). Together, they catalyze an essential two-step epimerization reaction that leads to the formation of decaprenylphosphoryl-D-arabinose (DPA), the only known source of arabinofuranosyl residues that are used by the mycobacterial arabinosyltransferases in the biosynthesis of important cell wall components such as arabinogalactan and lipoarabinomannan (10, 11, 39, 40, 42, 43, 45–52). The epimerization reaction catalyzed by the DprE1/E2 complex starts with decaprenylphosphoryl-β-D-ribose (DPR) as the initial substrate and proceeds through a decaprenylphosphoryl-D-2-keto-ribose (DPX) intermediate to give rise to DPA, and this is a two-step oxidation-reduction reaction (10, 11, 42, 45–52) (Fig. 4). First, DprE1 uses FAD as a cofactor to oxidize the 2′-hydroxyl group of DPR, generating a reduced flavin cofactor and DPX (10, 11, 42, 46, 48–51). The reduced FAD can be recycled to an oxidized form through the action of electron acceptors such as molecular oxygen or menaquinone (11, 46). Subsequently, DprE2 uses NADH or NADPH as a cofactor to reduce DPX to the final product, DPA (46–52).

Owing to the essential nature of the reactions catalyzed by DprE1/DprE2, many compounds have been developed to target either protein, although efforts have largely been focused on DprE1. The nitrobenzothiazinones (BTZs) were one of the first compounds reported to target DprE1 (10, 11, 40, 45, 48, 50). The BTZs work as mechanism-based inhibitors of DprE1, where the protein is both the activator of the prodrug and the target of the activated compound (Fig. 4). Here, DprE1 serves as a nitroreductase that uses its FADH_2_ prosthetic group to reductively activate the nitro group of the BTZs into a nitroso intermediate. The activated intermediate is an electrophile and is predicted to be susceptible to a nucleophilic attack by the thiol group of Cys387, an essential residue at the active site of DprE1. The electrophilic nitroso intermediate forms a covalent adduct with Cys387, irreversibly inhibiting the activity of the protein (10, 11, 40, 42, 45, 46, 50). Covalent inhibitors of DprE1 share the same mechanism of action as BTZs and are generally characterized by three properties: the presence of a nitro group, their dependence on the reductase activity of DprE1 for their activation into active intermediates, and lastly, their loss of inhibitory activity against Mtb mutants that have mutations in *dprE1* Cys387. There are several distinct covalent DprE1 inhibitors including dinitrobenzamides, trinitroxanthones, nitrobenzoquinoxalines, nitrotriazoles, nitrobenzothiazoles, and the more recently described, nitrofuranyl hydrazides (11, 12, 15, 40, 43, 45, 48, 53, 54).

Inhibitors of DprE2 have only recently been reported (51, 52), and this began with the work of Batt et al. who observed that the overexpression of DprE2 and not DprE1 in a whole-cell target-based screening reduced the potency of two nitrofuran-based compounds (52). They speculated that the compounds might be DprE2 inhibitors. However, when they followed up their observation with an *in vitro* biochemical assay for DprE2, they did not observe any inhibitory effect of the compounds on the enzymatic activity of DprE2. This was suggested to be due to the possibility that the compounds are prodrugs that need to be activated into a form that can interact with DprE2. Indeed, subsequent forward genetic selection proved this correct, with the implication of the cofactor F_420_ system as the activation machinery. However, the activating nitroreductase was never reported. In a later report by the same group, they showed that DprE2 is also a molecular target for the Ddn-activated pretomanid and delamanid (51). The activated nitroimidazole drugs were shown to form NAD adducts that inhibit the activity of DprE2 (Fig. 4). While the two studies from Batt et al. and Abrahams et al. are currently the only reports on DprE2 inhibitors (51, 52), it is intriguing that neither of them implicated the enzyme as a nitroreductase. As discussed previously, DprE2 works as a reductase to convert DPX to DPA using NADH or NADPH as a cofactor (47–52). Therefore, it is reasonable to argue that DprE2 might be able to reduce nitro-containing substrates. It is possible that as more DprE2 inhibitors are discovered, we will see nitro-containing compounds that require the reductase activity of the protein for their activation.

### Mrx2 (Rv2466c)

Rv2466c (DsbA/Mrx2) is a mycothiol-dependent cytosolic thioredoxin-like oxidoreductase that is directly induced by SigH as part of the bacterial response against oxidative stress (14, 44, 55–57). SigH also induces the expression of thioredoxin reductase/thioredoxin genes (*trxB2*/*trxC*) (55). During oxidative stress, the bacteria use these SigH-induced proteins to reduce disulfide bonds of different proteins, maintaining the cellular redox balance and protein conformations (44, 55–57). Notably, the mycothiol dependence of Rv2466c is critical to the protective activity of the protein against oxidative stress (14, 56, 57). Mycothiol is a low-molecular-weight pseudodisaccharide that is composed of a 1-d-*myo*-inosityl 2-amino-2-deoxy-α-d-glucopyranoside conjugated with *N*-acetylcysteine. The molecule is not found in eukaryotes, and its distribution seems to be limited to actinomycetes such as mycobacteria (58, 59). It is present in high levels in these actinomycetes, where it serves as the major low-molecular-weight thiol. This contrasts with most bacteria and eukaryotes that predominantly use glutathione, a tripeptide thiol, to maintain redox homeostasis inside the cells (57, 58, 60). Mycothiol is considered a functional analog of glutathione since it can mediate the same activities of the tripeptide molecule (60). The disruption of the multi-step biosynthetic machinery for mycothiol leads to enhanced susceptibility of the mycobacteria to oxidative stress (58–60).

**Fig 5:**
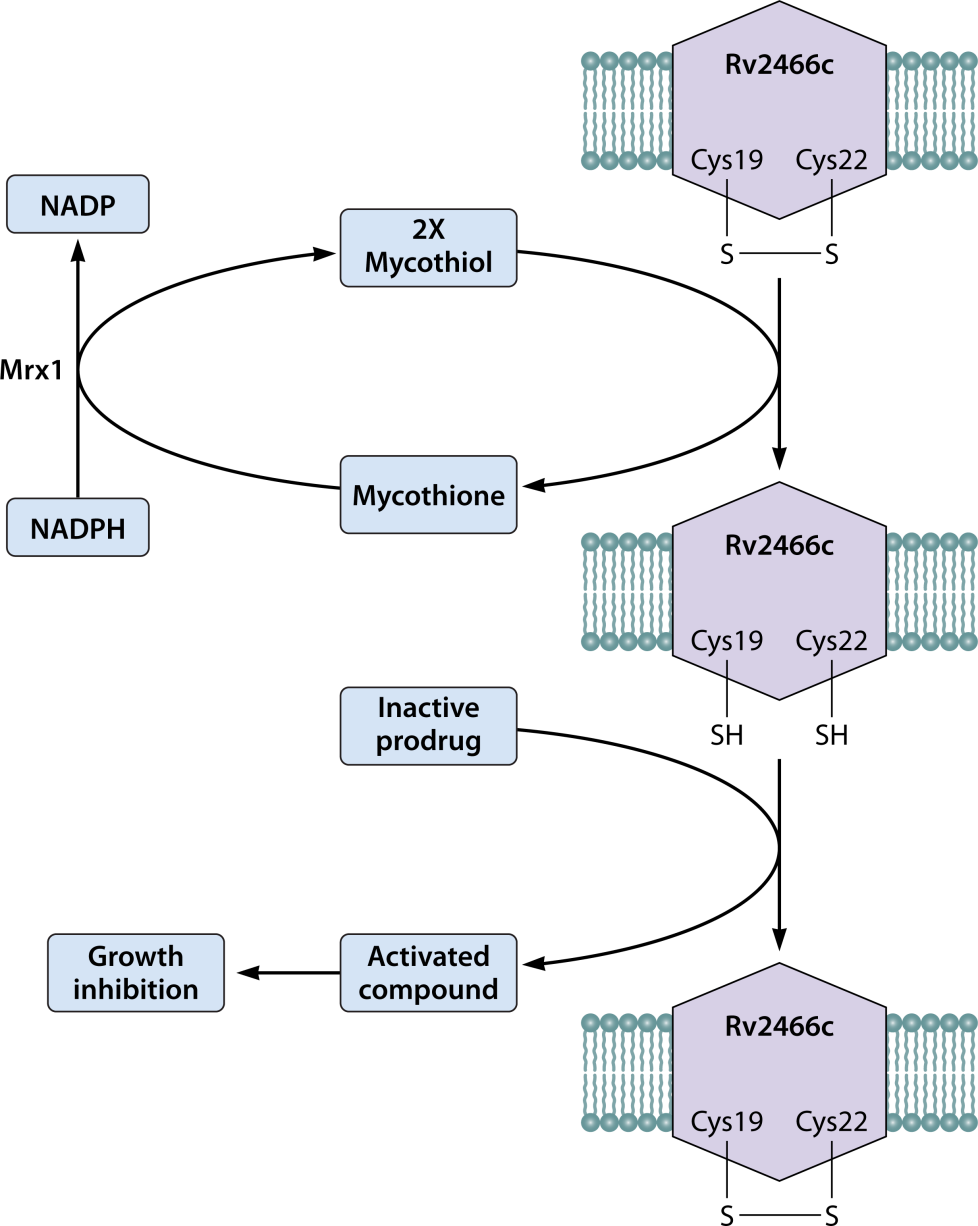
Activation mechanism of Rv2466c-dependent compounds. Two cysteine residues at the active site of Rv2466c form a disulfide bond that is broken down in a mycothiol-dependent manner. This generates a mycothione molecule that is recycled back to two mycothiol molecules through the catalytic activity of Mrx1, an enzyme that utilizes the reducing power of NADPH. The reduced Rv2466c enzyme can then activate nitro prodrugs into active metabolites that kill the bacteria. This also regenerates the oxidized form of the enzyme that can participate in multiple cycles of the reaction.

Rv2466c is a mycothiol-dependent nitroreductase that was first shown to reductively activate thienopyrimidine derivatives, with the mechanistic basis being worked out to be a series of dithiol-disulfide formations (44, 56, 57). Albesa-Jové et al. provided a structural model that suggests that the conformation of Rv2466c is controlled by its redox state, and this in turn, controls the catalytic activity of the protein (56). In the oxidized state, Cys19 and Cys22 at the active site of the protein form a disulfide bond with each other and trigger an inactive open conformation of the protein. Upon reduction, the protein switches to an active closed conformation that can catalytically activate thienopyrimidines. Upon reductive activation of thienopyrimidines, the Cys19-Cys22 disulfide bond at the active site of the enzyme is formed again. This leads to a local conformational change that opens the protein to release the activated prodrug. Taking this further, Rosado et al. worked out a biochemical model where Cys19 and Cys22 are in direct competition with each other (57). Cys22 promotes the formation of an intramolecular disulfide bond with Cys19, making the protein unable to activate the prodrug. This is akin to the oxidized inactive state that was proposed in the structural model of Albesa-Jové et al. (56), where Cys19 reacts with two mycothiol molecules, giving rise to a reduced protein and an outgoing mycothione molecule (Fig. 5). It is this reduced Rv2466c, through an initial nucleophilic attack by Cys19, that activates thienopyrimidines into active metabolites including nitric oxide species that kill Mtb (44, 56, 57, 61).

Mycothione, a molecule formed from the intramolecular disulfide linkage of two oxidized mycothiol molecules, can be recycled back to the reduced form through the action of mycothione reductase (Mrx1) with NADPH as an electron donor (57). Since Rv2466c is a mycothiol-dependent nitroreductase, it has been renamed as Mrx2 (57, 61, 62). While Rv2466c is considered a non-essential gene under normal laboratory conditions, it might play critical roles during the pathogenesis of the bacteria. This idea is driven by the conservation of a homologous gene in *M. leprae* (Fig. 1), a species that has undergone extensive genomic reduction and is presumed to retain only genes that are essential to its obligate intracellular physiology.

Besides thienopyrimidines, Rv2466c reductively activates nitrofuranylcalanolides (NFCs) into a fluorescent amine-based product, ANI (14). While NFCs have a high mycobactericidal activity, treatment of Mtb with a synthetic ANI shows a very weak cidal effect. This discrepancy in the mycobactericidal activity of the two compounds was proposed to be due to the poor cellular entry of ANI, although this remains to be tested. Alternatively, it is possible that ANI is not the bactericidal product of NFC and like most activated nitro compounds, the activated intermediates are too unstable to be reliably detected by existing tools. Nevertheless, the intrinsic fluorescence property of the activated NFC has been successfully applied toward the development of diagnostic assays for Mtb (14, 63, 64).

### Rv3368c

Rv3368c is an oxidoreductase that is conserved in different mycobacterial species (Fig. 1). It is upregulated in oxygen-starved non-replicating mycobacteria, pointing to a possible role in persistence (65). While Rv3368c is annotated as a nitroreductase, biochemical evidence to this effect is limited. Recently, Hong et al. showed that Rv3368c is possibly a nitroreductase that activates a cyanine-based nitro-containing probe for TB diagnosis (66). Here, the intrinsic fluorescence of cyanine is blocked by conjugating with a nitroaromatic ring. The reductive activity of Rv3368c is suggested to generate an amine derivative that allows the fluorescence of the cyanine probe to be detected. When the cyanine-nitroaromatic probe was further conjugated with trehalose, it allows for the specific labeling of live mycobacteria in clinical samples. Specifically, the trehalose in the cyanine-nitroaromatic probe is incorporated into the mycobacterial cell wall by actively replicating bacteria, allowing the probe to serve as a viability marker. Notably, this differs from standard diagnostic sputum smear reagents such as the fluorescent dye, auramine O, and the Ziehl-Neelsen staining that cannot differentiate between live and dead mycobacteria. Further biochemical analysis is needed to confirm the nitroreductase status of Rv3368c and to define the cofactor(s) needed for its activity. This will allow the development of Rv3368c-dependent diagnostic kits and possibly drugs for TB chemotherapy.

### Rv3131

Rv3131 is a *dosR*-regulated putative nitroreductase that is proposed to be part of the bacterial response to host-generated nitrosative stress during latent infection (67–69). It is immunogenic, stimulating the expression of proinflammatory cytokines (70). Due to its immunostimulatory property, Rv3131 has been considered as a potential vaccine candidate to protect against the hypervirulent Beijing Mtb strain (68, 71). Rv3131 is an FMN-bound protein that depends on NADPH for its oxidoreductase activity (72, 73), although its nitroreductase function is disputed (73). Recently, Dong et al. provided preliminary biochemical evidence on the nitroreductase function of Rv3131 (72). They showed that the protein uses NADPH to reductively activate metronidazole with two cysteine residues—Cys75 and Cys279—playing a role in this process. Further genetic studies could be used to decipher if Rv3131 is indeed the nitroreductase that activates metronidazole in Mtb. Metronidazole is a nitroimidazole-based drug that has been used in the treatment of different anaerobic infections (74–76). The low reduction potential of metronidazole ensures that it can only be reduced inside anaerobic organisms or hypoxic conditions. In such organisms or conditions, different systems such as the malate/pyruvate:ferredoxin oxidoreductases and hydrogenases reductively activate the drug (6, 74, 76, 77). Since Rv3131 is part of the 48-member DosRST regulon that is strongly upregulated in hypoxia-driven latent TB, it is possible that it might indeed be the nitroreductase that allows metronidazole to exert its antimycobacterial activity only in low-oxygen environment (72). Bioinformatic analyses suggest some similarity between Rv3131 and RdxA, a nitroreductase in *Helicobacter pylori* that is involved in the activation of metronidazole in the bacteria, reinforcing the possibility that Rv3131 is a metronidazole-activating nitroreductase in Mtb (72, 76). However, it cannot be ruled out the possibility that other nitroreductases might be the nitroreductase for metronidazole in Mtb, including the DosR-regulated protein Acg.

### Acg (Rv2032)

Rv2032 (Acg) is a monomeric FMN-bound protein that is induced by DosR (78, 79). Therefore, the gene is strongly upregulated during hypoxic conditions or during the infection of macrophages (69, 78, 79). Acg is generally classified as a nitroreductase (69, 78), although the evidence for this classification is contested. Support for the nitroreductase function of Acg comes from the work of Chauviac et al. who provided the first crystal structure of the protein (78). They showed that Acg has a structural fold that is reminiscent of classical nitroreductases. The protein can structurally superimpose with NfnB, a mycobacterial nitroreductase that is not found in Mtb. Going further, they showed that, like NfnB and many other nitroreductases, Acg uses FMN as a prosthetic group to accept or donate electrons. The FMN group can be reduced by dithionite in anaerobic conditions. Interestingly, Acg diverges from other FMN-bound nitroreductases in its inability to use NADH or NADPH as an electron source. Additionally, the Acg protein has a lid that may restrict the access of different substrates to the bound FMN pocket. This restrictive lid raises the question of which type of molecules can gain access to the binding pocket and if the protein even has any native nitroreductase activity. Evidence against the nitroreductase nature of Acg can be seen by the increased sensitivity of an *acg* knockout mutant to nitrofuran-based prodrugs (79). These drugs need to be reductively activated, and the fact that the mutant shows collateral susceptibility to the drugs suggests at least that the protein is not an activating enzyme for this nitro chemotype. However, it might also be that other nitro chemotypes can be reduced by Acg, and these were not tested in the study. In any case, Chauviac et al. proposed a model to explain the increased susceptibility of the *acg* mutant to the nitrofurans (78), suggesting that Acg might be sequestering the FMN cofactor needed by other nitroreductases, serving as a storage site for the cofactor. The inactivation of Acg will increase the cellular availability of the FMN cofactor, and this can be used by other nitroreductases to reductively activate the nitrofuran prodrugs. Alternatively, it can be speculated that Acg might be functioning as an NfnB-like nitroreductase that inactivates the nitrofurans, hence the increased susceptibility of the mutants to the compounds.

## MOLECULAR TARGETS OF NITRO-CONTAINING COMPOUNDS

With the exception of the DprE1 mechanism-based inhibitors, where DprE1 is both the activator and target, the molecular targets of most nitro-containing compounds are multifactorial. The reductive activation of the aromatic nitro groups of these compounds is proposed to release radical nitrogen species that rarely have only a single cellular target (5, 6). These species damage different cellular components including DNA, RNA, and proteins (77). Many nitro-containing compounds are effective against both active and non-replicating persistent Mtb. This contrasts with many TB drugs that are only active against actively replicating Mtb. The potency of nitro prodrugs against active and non-replicating Mtb may be attributed to the ability of the compounds to target mycolic acid biosynthesis and respiration under different physiological conditions. This is a hypothesis that was primarily built from the transcriptional profiling of pretomanid (5, 35) but can be generalized to other nitro-containing compounds (9, 80). Part of the activities that occur in an actively replicating Mtb is the biosynthesis of mycolic acids (5). In such organisms, nitro compounds exert their antitubercular activity by inhibiting different enzymes involved in the biosynthesis of mycolic acids, and some of these effects have been biochemically validated. For instance, JSF-2019, a nitrofuranyl triazine, has been shown to be a direct inhibitor of InhA, an enzyme involved in the FAS-II pathway of mycolic acid biosynthesis (9). Pretomanid reduces the levels of ketomycolates and allows the accumulation of the precursor, hydroxy-mycolates, possibly by its direct inhibition of FHMAD, an enzyme that converts hydroxy-mycolic acids into keto-mycolic acids (24, 30, 35). As mentioned previously, pretomanid and delamanid have recently been shown to be inhibitors of DprE2, another protein involved in the biosynthesis of the mycobacterial cell envelope (51). Despite the subtle differences in the ability of nitro compounds to inhibit different genes involved in mycolic acid biosynthesis, a uniting feature seen in the transcriptional profiling of most nitro compounds is the upregulation of the *iniBAC* operon (5, 9, 80). This operon is typically upregulated by inhibitors of mycobacterial cell wall biosynthesis (5).

On the other hand, cell envelope biosynthesis is limited in non-replicating Mtb, making drugs such as isoniazid, ethambutol, DprE1 inhibitors, and other drugs that target cell envelope biosynthesis ineffective against non-replicating Mtb (5, 10, 15, 35). The minimal basal transcriptional state of non-replicating Mtb also makes it challenging to conduct transcriptional studies in such cells (5). However, transcriptional profiling in aerobically growing Mtb coupled with biochemical studies in non-replicating cells has led to a model where the nitro compounds primarily inhibit respiratory activities in non-replicating Mtb (5, 7, 9, 80). In aerobic conditions, the released nitric oxide can be detoxified by molecular oxygen; but in hypoxic conditions, the free nitric oxide species are sufficient to drive the antimycobacterial activities of the compounds (61, 80). Non-replicating mycobacterial cells show some levels of respiration that are needed to maintain critical cellular processes such as membrane potential, and the bactericidal activity of the nitro compounds can be explained by the ability of the released nitric oxide to serve as an electron sink (5, 7, 28, 62, 77, 80). Like the respiratory inhibitor, potassium cyanide, the released toxic nitric oxide is proposed to interact with cytochromes or cytochrome oxidases in the electron transport chain, although this interaction has not been elucidated for the nitro compounds (5, 7). This hypothesis is inferred from the differential expression of sentinel respiratory genes such as the *cydABDC* operon that encodes cytochrome *bd* oxidase, the nitrate reductase *narGHIJ*, the type 1 NADH dehydrogenase *rv1854c*, and cytochrome genes such as *rv0327c* and *rv0136* among others (5, 61, 80). Biochemically, this respiratory poisoning normally manifests as a rapid decrease in the intracellular concentrations of ATP and a change in the redox status of the non-replicating Mtb (5).

Nitro compounds can also target genes that are clearly far from pathways related to respiration or mycolic acid biosynthesis. For example, Mori et al. used a combination of genetics and click chemistry to demonstrate that TP053, a DsbA-activated thienopyrimidine, directly interacts with Rv0579, a non-essential mycobacterial protein that is proposed to have RNase activity (62). The physiological activities of Rv0579 are still unclear, although it is suggested to be involved in RNA metabolism. Thus, in addition to other mechanisms, TP053 may be interrupting the metabolism or turnover rate of the mycobacterial RNA pool by targeting Rv0579.

## CONCLUDING REMARKS

The lack of inhibitory activity of most nitro prodrugs discussed in this paper against non-mycobacterial (or non-actinobacterial) species has been attributed to the species-specificity of the mycobacterial nitroreductases. However, we note that this hypothesis might be overlooking other factors that play a role in the cellular pharmacodynamics of an antimicrobial agent. These factors include, but are not limited to, cellular permeability, efflux systems, drug metabolism and modifications, and in the specific case of prodrugs, amino acid sequence differences in the activating enzyme and the presence/absence of a suitable cofactor that is used by the activation machinery. For instance, an ortholog of Ddn exists in *Staphylococcus aureus*, but Ddn-dependent drugs such as pretomanid or HC2210 are inactive against the bacteria (15). This inactivity might be caused by poor sequence conservation of the *S. aureus* ortholog (about 47% amino acid sequence homology). Other bacteria such as *Escherichia coli*, *Proteus vulgaris*, and *Pseudomonas aeruginosa* lack a Ddn ortholog and predictably show resistance to Ddn-dependent drugs (15). Overall, detailed biochemical studies are needed to further understand the species-specificity of mycobacterial nitroreductases.

The diversity of small molecules that kill mycobacteria and are dependent on nitroreductases provides strong motivation to continue defining the biological and biochemical functions of this class of enzymes. Because resistance to nitro prodrugs evolves mostly from mutations in nitroreductases and their cofactors, understanding the functions of this enzyme class may provide new approaches on combating the evolution of drug resistance to nitro-containing compounds. The native activity of several nitroreductases enables the pathogen to survive harsh conditions such as oxidative stress. Therefore, it stands to reason that the genetic disruptions of these nitroreductases or the biosynthesis machinery for the cofactors may make the pathogen to be more susceptible to the stress agent. For instance, F_420_-deficient or *fgd* mutants are known to be hypersusceptible to oxidative stress (18, 22). Of note is that F_420_-deficient mutants are also hypersensitive to oxidative stress-elevating drugs such as isoniazid, moxifloxacin, and clofazimine (18), highlighting the concept of collateral sensitivity. Hence, these drugs can be used in combination with F_420_-dependent compounds for TB treatment. This will ensure that resistance to these F_420_-dependent compounds that are conferred by the disruptions in the biosynthesis of F_420_ reduction or recycling machineries will come with a collateral cost in terms of increased susceptibility to these drugs. Interestingly, a recent report by Waller et al. (81) offered a conflicting report for F_420_-deficient or *fgd* mutants and their hypersusceptibility to clofazimine. They showed that these mutants have a low-level resistance to clofazimine. In the same study, Waller et al. reported pretomanid as hyper-active against mutants that are resistant to either rifampicin, levofloxacin, isoniazid, Q203, or bedaquiline. This finding further supports the potential durability of the 4-month bedaquiline-pretomanid-linezolid regimen since bedaquiline-resistant Mtb exhibits collateral sensitivity to pretomanid. With the proven clinical success of pretomanid and delamanid against Mtb, continued efforts to develop new drugs dependent on nitroreductases are anticipated, driving the need to better understand the biochemical and physiological functions of mycobacterial nitroreductases.

## SUPPLEMENTAL MATERIAL

Supplemental figures jb.00326-24-s0001.pdf Fig. S1 and S2.

ASM does not own the copyrights to Supplemental Material that may be linked to, or accessed through, an article. The authors have granted ASM a non-exclusive, world-wide license to publish the Supplemental Material files. Please contact the corresponding author directly for reuse.
