# Supplementary material for: Functions of nitroreductases in mycobacterial physiology and drug susceptibility
Source: J Bacteriol. 2025 Jan 8;207(2):e00326-24. doi: 10.1128/jb.00326-24 (PMC11841060; doi:10.1128/jb.00326-24)
Supplement: Supplemental figures — Fig. S1 and S2. [file jb.00326-24-s0001.pdf]

Supplemental Figures

Supplemental Figure 1. Structures of selected molecules in discussed in the review. A. Cofactor F420-dependent compounds**. B.** DprE1 mechanism-based inhibitors. **C.** Other nitro-based compounds. **D.** Cofactors used for prodrug activation.

Supplemental Figure 2. Multiple sequence alignment of mycobacterial Ddn orthologs. All amino acid sequences were compared against Mycobacterium tuberculosis Rv3547 (also known as Ddn). Except for Rv3547, all the identifiers in bracket represent the NCBI reference sequence identifier. ClustalOmega in SnapGene was used for the multiple sequence alignment of the Ddn orthologs.

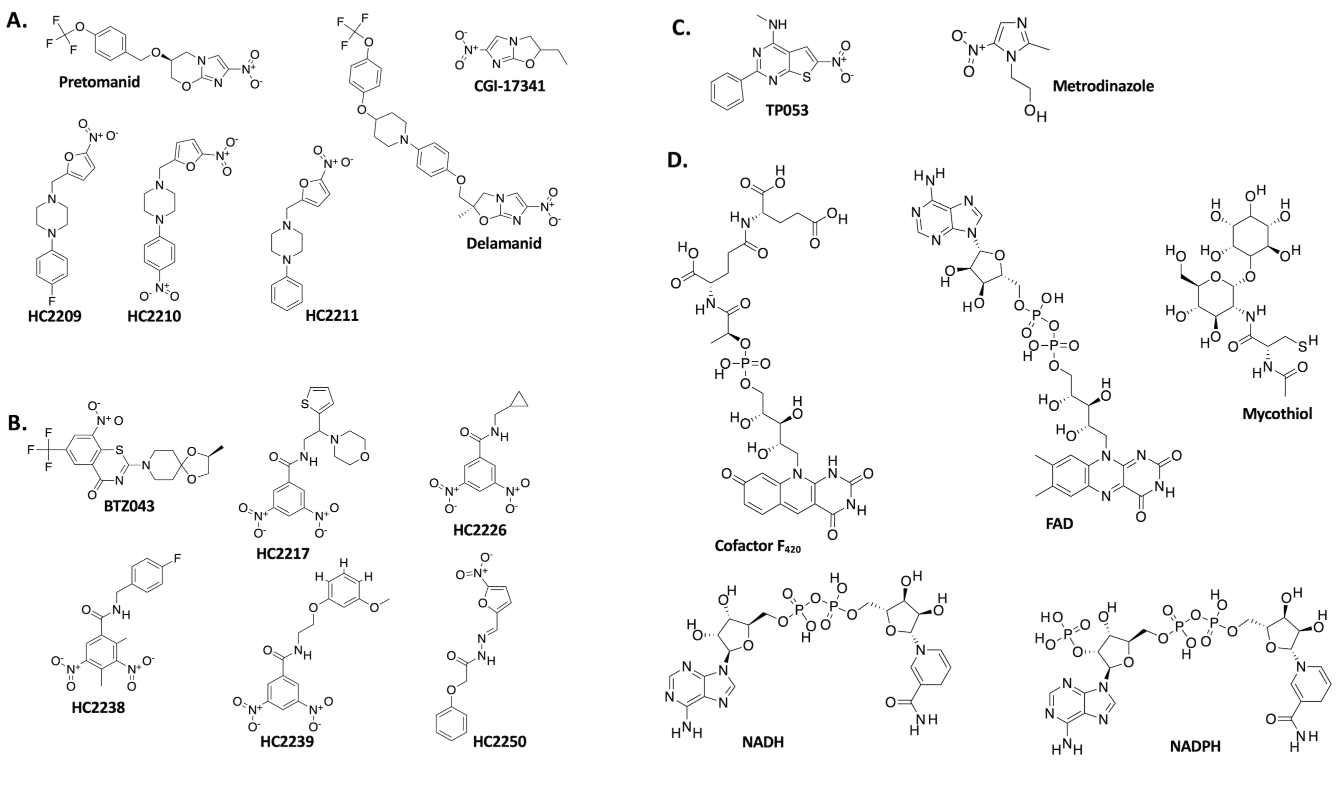

Supplemental Figure 1

```
*  Mycobacterium tuberculosis H37Rv (Rv3547)      -----------MPKSPPRFLNSPLSDFFIKWMSRINTWMYRRNDGEGLGGTFQ--------KIPVALLTTTGRKTGQPRVNPLYFLRDGGRVIVAASKGGAEKNPMWYLN 91
*  Mycobacterium marinum (WP_012396559.1)         -----------MPKSPPRFLNSPFTDFFIKWMSRINTFMYRRNGGEGLGGTFQ--------KIPVALLTTTGRKTGEPRVSPLYFHRDGDRVIVAASKGGSAKNPMWYLN 91
*  Mycobacterium kansasii (WP_023368421.1)        -----------MPKSPPRFLNSPFTDVLIKWMSRLNTWMYRRNGGEGLGGTFQ--------KIPVALLTTTGRKTGQPRVSPLYFLRDGDRVIVAASKGGADKNPMWYLN 91
*  Mycobacterium xenopi (WP_003923123.1)          --------M--PSKSPPRFLNSPFTDFVIKWMSRVNTFLYRRGGGEGLGGTFQ--------KRPVALLTTTGRKTGQPRVSPLLYLRDGDRVILVASRGGSAKNPMWYLN 92
** Mycobacterium smegmatis (WP_058127102.1)       ----MADTSRPLNAKQLERLNAKSTGTLIKWMSRFQTFLFKTTNG-KLGNKFLRG-------TEVGILTTIGRKSGEPRDTPLLFLQEGRRIVLVASQGGRATNPMWYLN 98
** Mycobacterium ulcerans (WP_156091826.1)       -----------MPKSPPRFLNSPYTDFFIKWMSRINTFMYRRNDGEGLGGTFQ--------KIPVALLTTTGRKTGEPRVSPLYFHRDGDRVIVAASKGGSAKNPMWYLN 91
** Mycobacterium avium (ABK69399.1)              -------------------MNSPMTGFFIKWMSRVNTWMYRVSRG-KWGGTFQ--------KRPVALLTTTGRKTGQPRVSPLLYLREGDRVILVASQGGRDKHPLWYLN 82
** Mycobacterium intracellulare (WP_064935907.1) -----------MPKPKPRALNSPWVAFIIKWMAKSNAWIYRRSNG-KFGGTFQ--------KAPVALLTTTGRKTGEPRVSPLLYLREGDRVILVASKGGSDKHPLWYLN 90
** Mycobacterium gordonae (WP_205875021.1)       -----------MPKSRPRFQDSPLTDHFIKLMSRLNTWLYRRNGGEGLGGTFQ--------KIPVALLTTTGRKTGEPRVSPLYFLRDGDRVIVAASKGGAAKNPMWYLN 91
** Mycobacterium chelonae (WP_078354316.1)       MSDGLAASG--SSANAPAQLNSELAGKLIKWMTAANVWLYQRTDG-RLGGKWRVGA-AFPWGIPVLLITTIGRKTGQKRLSALLYLPDGDKIVLVASQGGRDANPAWYLN 106
** Mycobacterium fortuitum (WP_141142891.1)      ----MANTPRPLSPKQVERLNAKSTDTAIKWMSRAQTWIFKKTGG-RIGDKFLRG-------AEVGILTTIGRKSGEERDSPLLFLQEGKRIVLVASQGGRATNPMWYLN 98
** Mycobacterium scrofulaceum (WP_083178703.1)   -----------MPKPKPRSLNAPWVGFIIKWMAKGNTWIYRLSNG-RFGGTFQ--------NAPVALLTTTGRKTGEPRVSPLLYLREGNRVVLVASKGGSDKHPLWYLN 90
** Mycobacterium gilvum (ABP43987.1)             ----MTKAPKPLTPKQVESLNSNAVGTGIKWMSKLNTWAYKATGG-RLGAKWRGGSNRFSAPPPVGILTTIGRKSGEPRESPLLFLREGDRIVLVASQGGRATNPMWYLN 105
** Mycobacterium abscessus (WP_079626321.1 )     ----MTAPG--SSANAPAQLNSEFAGKVIKWMTAVNVWLYQRTDG-RLGGKWRVGA-AFPWGIPVLLLTTTGRKTGQQRLSALLYLSDGDKVVLVASQGGRASNPAWYLN 102

Mycobacterium tuberculosis H37Rv (Rv3547)      LKANPKVQVQIKKEVLDLTARDATDEERAEYWPQLVTMYPSYQDYQSWTDRTIPIVVCEP- 151
Mycobacterium marinum (WP_012396559.1)         LKANPKVGVQIKKEVLDLTARDATDEERARYWPKLVDMYPSYEDYQSWTDRTIPIVVCEP- 151
Mycobacterium kansasii (WP_023368421.1)        LKANPKVSVQIKKEILDLTARDATDEERAKYWRQLVDMYPTYEDYQSWTDRTIPIVVCEP- 151
Mycobacterium xenopi (WP_003923123.1)          LKANPQVQVQIKKEVLNLTARDATEEERAMYWPQLVAMYPSYQDYQAWTDRTIPIVVCEP- 152
Mycobacterium smegmatis (WP_058127102.1)       LKANPKVTFQTKSEKLALVAREATDAERDEYWPKLDAMYPDFANYRSYTDRKIPIVICDPA 159
Mycobacterium ulcerans (WP_156091826.1)        LKANPNVGVQIKKEVLDLTARDATDEERARYWPKLVDMYPSYEDYQSWTDRTIPIVVCEP- 151
Mycobacterium avium (ABK69399.1)               LKANPKVSVQIKDEVLQLQARDATPEEREQYWPKLVAMYPSFDDYQSWTDRVIPVVICDP- 142
Mycobacterium intracellulare (WP_064935907.1)  LKANPKVSVQIKDEVLHLRARDATEAERAEYWPKLDAMYPSFDDYRSWTDRVIPVVICDP- 150
Mycobacterium gordonae (WP_205875021.1)        LKANPQVQVQIKKEVLDLTARDATDEERARYWPQLVDMYPSYEDYQSWTDRTIPIVICAPA 152
Mycobacterium chelonae (WP_078354316.1)        LKANSEVTVQIKGDIRTMTARTATDEERAYYWPKLVELYADFAKYQSYTTRKIPVVVLEP- 166
Mycobacterium fortuitum (WP_141142891.1)       LVANPRVKFQTKRETLELVARDATDAERDVYWPKLDAMYADFANYRSYTDRKIPIVICDPA 159
Mycobacterium scrofulaceum (WP_083178703.1)    LKANPKVSVQIKDEVLQLQARDATEAERAEYWPKLDAMYPSFDDYRSWTDRVIPVVICDP- 150
Mycobacterium gilvum (ABP43987.1)              IQANPKVTFQVKNEKLTLVARDATDAERDEYWPKLDAIYPDFVNYRTYTDRKIPILICDPA 166
Mycobacterium abscessus (WP_079626321.1 )      LKANSEVTVQIKGDIRTMTARTATDAERAYYWPKLVELYADFDKYQSYTTRQIPVVILEP- 162
```

* = susceptible to pretomanid

** = resistant to pretomanid

Supplemental Figure 2
